# Broadening of the T-Cell Repertoire to HIV-1 Gag p24 by Vaccination of HLA-A2/DR Transgenic Mice with Overlapping Peptides in the CAF05 Adjuvant

**DOI:** 10.1371/journal.pone.0063575

**Published:** 2013-05-17

**Authors:** Karen S. Korsholm, Ingrid Karlsson, Sheila T. Tang, Lea Brandt, Else Marie Agger, Claus Aagaard, Peter Andersen, Anders Fomsgaard

**Affiliations:** 1 Department of Infectious Disease Immunology, Statens Serum Institut, Copenhagen, Denmark; 2 Department of Virology, Statens Serum Institut, Copenhagen, Denmark; Deakin University, Australia

## Abstract

Induction of broad T-cell immune responses is regarded as critical for vaccines against the human immunodeficiency virus type 1 (HIV-1) which exhibit high diversity and, therefore, focus has been on inducing cytotoxic CD8 T-cell responses against the more conserved parts of the virus, such as the Gag protein. Herein, we have used the p24 protein which contains a range of conserved T-cell epitopes. We demonstrate that a vaccine of HIV-1 subtype B consensus group-specific antigen (Gag) p24 protein with the CD8-inducing liposomal cationic adjuvant formulation (CAF) 05, induces both CD4 and CD8 T-cell responses in CB6F1 mice. The adjuvanted vaccine also induced functional antigen-specific cytotoxicity *in vivo*. Furthermore, we found that when fragmenting the Gag p24 protein into overlapping Gag p24 peptides, a broader T-cell epitope specificity was induced in the humanized human leukocyte antigen (HLA)-A2/DR-transgenic mouse model. Thus, combining overlapping Gag p24 peptides with CAF05 appears to be a promising and simple strategy for inducing broader T-cell responses to multiple conserved epitopes which will be relevant for both prophylactic and therapeutic HIV-1 vaccines.

## Introduction

Despite increasing availability of antiretroviral treatment (ART), the acquired immunodeficiency syndrome (AIDS) is still the underlying cause for more deaths worldwide than any other infectious disease [1]. Thus, vaccination, both prophylactic and therapeutic, is an important alternative or supplement to conventional treatment and in addition, it is the only intervention that can provide immunological memory. Whereas induction of antibodies against the envelope protein (Env) is required for prophylactic vaccines, T-cell responses are important for immune control of HIV-1 replication [2], [3], [4] and different T-cell activating therapeutic vaccines are being developed and tested [5], [6], [7].

It is debated which are the most effective antigens to target for T-cell based HIV-1 vaccines. Although broad immunity to multiple HIV-1 proteins is thought to be more efficacious [8], [9], the most effective HIV-1 antigen to target for T-cell based therapeutic vaccination seems to be the precursor polyprotein, Gag [10], [11], [12], which is proteolytically processed into the viral structural matrix and capsid proteins. This may partly be due to the relatively high conservation of Gag but also the early presentation of Gag peptides from the incoming virus on major histocompatibility complex class I (MHC-I) on infected cells before viral integration, protein synthesis and MHC-I down-regulation by the negative regulatory factor (Nef) occur [13]. Moreover, the ability to mount multiple simian immunodeficiency virus (SIV) Gag-specific CD8 T-cell responses correlates with reduction of chronic phase viremia [13]. In studies using peptide-pulsed autologous peripheral blood mononuclear cell (PBMC) immunotherapy in SIV-infected macaques, overlapping peptides from SIV Gag (OPAL) or from the whole SIV proteome was found to be highly immunogenic and significantly lowered viral load if administered under ART [8], [10], [14], [15]. The SIV control induced by therapeutic vaccination showed no advantage in virus control using peptides spanning all 9 SIV proteins in comparison to just overlapping Gag peptides [10].

The use of professional antigen presenting dendritic cells to deliver HIV-1 immunogens has led to strong immunogenicity in macaque and human studies albeit with varying viral effects [16], [17]. However, adjuvanted subunit vaccines have been shown to promote long-term protective immune responses and would be even simpler, eliminating the need for *ex vivo* procedures. Thus, we have recently used HIV-1 CD8 minimal cytotoxic T lymphocyte (CTL) epitope peptides in the cationic adjuvant formulation (CAF) 01 in ART-naïve HIV-1 infected individuals to induce T-cell responses to immune subdominant epitopes infrequently targeted during chronic HIV-1 infection ([18], Roman et al., manuscripts in preparation). Such immunity to subdominant HIV-1 T-cell epitopes has been suggested to be of importance for virus control [4], [19]. However, in contrast to minimal CTL epitopes, which are HLA-specific, overlapping peptides can potentially overcome the HLA diversity, especially if also subdominant immune responses to conserved epitopes can be induced.

For a CTL subunit vaccine approach based on protein or overlapping peptides to be successful a good CD8 T-cell promoting adjuvant has to be included because the peptides should be delivered to antigen-presenting cells and properly processed for presentation on MHC-I. This kind of adjuvant should therefore promote cross-presentation of exogenously delivered antigens and priming of specific CD8 T cells. However, such an adjuvant is currently not commercially available. Therefore, the CAF01 adjuvant, which is both safe and immunogenic in humans [20], has been refined to enhance CD8 T-cell induction by incorporation of the Toll-like receptor (TLR) 3 ligand, Polyinosinic-polycytidylic acid (Poly(I:C)), into the liposomal formulation [21], [22].

We studied the T-cell responses in CB6F1 mice using whole Gag p24 protein delivered in either the CAF01 or the CAF05 adjuvant. When using the CAF05 adjuvant we achieved high CD4 and CD8 T-cell responses that were cytotoxic *in vivo*. The concept was then expanded to overlapping Gag p24 peptides (OLGa) in an attempt to maximize and broaden the CD8 T-cell response against Gag p24. This was done in HLA-A2/DR transgenic mice to address human-relevant T-cell epitopes [23]. We found that OLGa in CAF05 resulted in both a higher and broader T-cell response when compared with whole Gag p24 protein as the antigen.

## Materials and Methods

### Synthetic Gag p24 Peptides

A panel of 55 15-mer peptides with 11-aa overlap, representing the whole Gag p24 protein based on consensus HIV-1 sequence clade B, was provided by National Institutes of Health AIDS Research and Reference Reagent Program and NIBSC. Peptides were dissolved at a concentration of 4 mg/ml in 10 mM Tris buffer, pH 7.4 containing 4% dimethyl sulfoxide (DMSO). One peptide was impossible to solubilize and was therefore excluded from the experiments (ARP7111.77, sequence: RAEQASQEVKNWMTE). The AMQMLKETI peptide and peptides corresponding to peptide 16 and 17 of the peptide panel (see Table 1) where synthesized by Genscript.

**Table 1:** Peptide pool matrix.

Pool^1^	1	2	3	4	5	6	7	8
**9**	1	2	3	4	5	6	7	8
**10**	9	10	11	12	13	14	15	16
**11**	17	18	19	20	21	22	23	24
**12**	25	26	27	28	29	30	31	32
**13**	33	34	35	36	37	38	39	40
**14**	41	42	43	*NA* 2	45	46	47	48
**15**	49	50	51	52	53	54	55	

1 Gag p24 15-mer peptides ARP7111.34 to ARP7111.88 were numbered from 1–55 and assigned to 15 pools according to the matrix.

2 *NA*, not applicable.

### Recombinant Expression and Purification of HIV Gag p24

The amino acid sequence of the HIV-1 clade B consensus Gag p24 protein was: PIVQNLQGQMVHQAISPRTLNAWVKVVEEKAFSPEVIPMFSALSEGATPQDLNTMLNTVGGHQAAMQMLKETINEEAAEWDRLHPVHAGPIAPGQMREPRGSDIAGTTSTLQEQIGWMTNNPPIPVGEIYKRWIILGLNKIVRMYSPTSILDIRQGPKEPFRDYVDRFYKTLRAEQASQEVKNWMTETLLVQNANPDCKTILKALGPAATLEEMMTACQGVGGPGHKARVLA. The encoding gene was codon optimized for expression in *Escherichia coli* and inserted into the T7 expression vector pGS-21a (Genscript, Piscataway, NJ). In this process an N-terminal 6 x His-tag was added to the open reading frame to facilitate purification. Two separate cultures of *E. coli* BL21-AI (Life Technologies, Denmark) harboring the recombinant Gag p24 expression vector was grown in 6 L LB medium containing 100 µg/mL ampicillin. At OD_600_ ∼ 0.5 the cultures were induced with 0.2% (w/v) arabinose for 4 hours. Bacteria were pelleted and the outer membrane lysed using mild detergents according to the manufacturer recommendations (BPER™, Pierce, Rockford, IL) and the Gag p24 containing supernatant was dialyzed against 8 M urea, 100 mM Na_2_PO_4_, 10 mM Tris-HCl pH 8.0 (buffer A). After clearing by centrifugation, soluble protein was applied and bound to a 20 mL metal affinity column (Talon Superflow™, GE Healthcare, Denmark) pre-equilibrated in buffer A. Bound protein was washed 5 times with 2 column volumes (CV) of buffer, alternating between 10 mM Tris-HCl pH 8.0, 60% isopropanol, and 50 mM NaH_2_PO_4_ pH 8.0 before being eluted in 5 mL fractions in buffer A supplemented with 150 mM imidazole. All protein containing fractions were analyzed on Coomassie blue stained sodium dodecyl sulfate polyacrylamide gel electrophoresis (SDS-PAGE) gels and fractions with the highest amount of recombinant protein relative to contaminants were collected, pooled and dialyzed against 3 M urea, 10 mM Tris-HCl pH 8.5 before being applied to a 5 mL anion-exchange column (HiTrap MonoQ™, GE Healthcare, Denmark). After washing with 5 CVs of application buffer, Gag p24 was eluted using a linear gradient from 0 to 1 M NaCl over 40 CVs before being dialyzed against 0.01% acetic acid. Protein concentration and final yield was determined by a fluorescent based quantification kit (NanoOrange™ Protein Quantitation Kit, Life Technologies, Denmark) using bovine serum albumin as standard. The final yield for the two parallel purification of Gag p24 was 15 mg and 12.5 mg with a concentration of 1.6 mg/ml and 1.2 mg/ml, respectively.

### Adjuvant Preparation

The CAF01 adjuvant consists of Dimethyldioctadecylammonium bromide (DDA) (250 µg/dose) combined with trehalose dibehenate (TDB) (50 µg/dose) prepared in 10 mM Tris-buffer by the thin film method described elsewhere [24]. The CAF05 adjuvant consists of CAF01 which has been combined with Poly(I:C) (50 µg/dose). CAF05 was prepared based on the CAF01 adjuvant by gradual addition of Poly(I:C) in small volumes by means of vortexing followed by sonication in a water bath at 60°C.

### Animal Experiments

#### Ethics

The CB6F1 animal experiments were approved by the Danish Council for Animal Experiments and carried out in accordance with EU Directive 2010/63/EU for animal experiments. All protocols using HLA-A2/DR-transgenic mice were reviewed and approved by the Panum Institute, University of Copenhagen institutional animal care and use committee according to the Animal Experimentation Act of Denmark and European Convention ETS 123 (Protection of Vertebrate Animals used for experimental and other scientific purposes). All efforts were made to minimize suffering of the animals.

#### CB6F1 mice

Female CB6F1 mice (first generation offspring of a cross between female BALB/c and male C57BL/6) were purchased from Harlan (Horst, The Netherlands) animal care was in accordance with institutional guidelines. The mice were allowed free access to water and food and they were 8–10 weeks old at experiment initiation.

#### HLA-A2/DR-transgenic mice

The humanized HLA-A2.1−/HLA-DR1-transgenic H-2 class I−/class II-knock-out mice [23] were kindly provided by F.A. Lemonnier, Institut Pasteur, Paris, France, and were bred in the animal facility at The Panum Institute, University of Copenhagen. These mice express a transgenic mono chain histocompatibility class I molecule in which the C-terminus of the human ß_2_-microglobulin (ß_2_m) is covalently linked to the N-terminus of a chimeric heavy chain (HLA-A2.1 α1-α2, H-2D^b^ α3-transmembrane and intracytoplasmic domains) [23]. HLA-A2/DR-transgenic mice are homozygous for the transgene, and H-2D^b−/−^ and ß_2_m^−/−^ double knock out, respectively. In addition these mice lack cell surface expression of class II molecules (H2 IAβ^b^) which are replaced with the human HLA-DRA*0101 and HLA-DRB1*0101 molecules.

#### Immunizations

The vaccines were prepared as follows: Gag p24 protein (5 or 20 µg/dose) or a mixture of the 54 overlapping peptides (2 µg/peptide/dose) was mixed with the indicated adjuvants in 10 mM Tris buffer containing sucrose to make the vaccine isotonic (final concentration: 9% sucrose). The vaccines were left at room temperature for 30 minutes with intermittent mixing to allow for proper surface adsorption of the antigens to the liposomes. As controls a group of mice receiving a vaccine without adjuvant and a group of unimmunized naïve mice were included. The mice were treated with the analgesic Temgesic and immunized intraperitoneally with 200 µl of vaccine that had been pre-heated to body temperature at weeks 0, 2 and 4.

#### Splenocyte purification

The mice were killed by cervical dislocation and single-cell suspensions of spleen cells were obtained by passage of the spleens through a 70 or 100 µm cell strainer (BD, Albertslund, Denmark). Cells were subsequently washed twice with RPMI 1640 (Gibco Invitrogen, Carlsbad, CA, USA) and cultured in complete RPMI (RPMI 1640 supplemented with 10% (v/v) heat-inactivated fetal bovine serum, 5×10^6^ M β-mercaptoethanol, 1% (v/v) penicillin–streptomycin, 1% (v/v) sodium pyruvate, 1 mM L-glutamine, and 10 mM HEPES) in a humidified atmosphere at 37°C, 5% CO_2_. Remaining cells were frozen in RPMI 1640 supplemented with 60% FBS and 20% DMSO.

#### Enzyme-linked immunosorbent assay (ELISA)

Three weeks after the final immunization splenocytes were cultured for 4 days at a density of 2×10^5^ cells/well in round-bottomed 96-well plates (NUNC, Roskilde, Denmark). The cells were re-stimulated with the indicated amounts of antigen or peptide. Wells containing medium alone or 25 µg/ml of phorbol 12-myristate 13-acetate (PMA) and 1 µg/ml of ionomycin (Sigma–Aldrich, St. Louis, MO, USA) were included as negative and positive controls, respectively. After 4 days, the supernatants were collected and interferon (IFN)-γ production was quantified by a standard ELISA protocol. Briefly, purified rat anti-mouse IFN-γ (BD Biosciences, San Jose, CA,USA) was used as capture and biotin-conjugated rat anti-mouse IFN-γ (BD Biosciences) was used as detection antibodies, respectively, followed by horse-radish peroxidase (HRP)-conjugated streptavidin (BD Biosciences) and TMB Plus Ready-to-use substrate (Kem-En-Tec, Taastrup, Denmark). The reaction was stopped at the optimal color development with 0.2 M H_2_SO_4_, and absorbance was read at 450 nm with wavelength correction at 570 nm to correct for optical imperfections in the plates including air bubbles.

Detection of anti-Gag p24 antibodies in the sera from immunized mice was also done by ELISA. In brief, 96-well MaxiSorp plates (Nunc, Denmark) were coated with 0.5 µg/ml of Gag p24 in 15 mM Na_2_CO_3_, 35 mM NaHCO_3_ (pH 9.7), overnight at 4°C. The plates were blocked in phosphate-buffered saline (PBS) containing 2% (w/v) skimmed milk and sera were added in serial dilutions. Specific antibodies were detected following incubation with rabbit anti-mouse IgG1 or IgG2a conjugated to horseradish peroxidase (Zymed/Invitrogen). Development and absorbance reading was done as described above.

#### Intracellular cytokine staining

Splenocytes were rested at 4°C over night and then stimulated at a concentration of 1×10^7^ cells/ml in V-bottom 96-well plates with 10 µg/ml of the overlapping Gag p24 peptides used for immunizations for 1 hour at 37°C and 5% CO_2_. Then brefeldin A (10 µg/ml, Sigma-Aldrich, Denmark) was added and the cells were incubated for another 7 hours at 37°C in a Thermoplate Plus Heatblock (Eppendorf, Hørsholm, Denmark). After stimulation, the cells were washed in PBS with 1% fetal bovine serum (FBS) and surface-stained with 1 µg/ml of CD4:APC-eFlour780, CD8α:PerCp-Cy5.5 and CD44:FITC (all from eBioscience) for 30 minutes at 4°C in the dark. The cells were subsequently fixed and permeabilized using the Cytofix/cytoperm kit (BD Biosciences) according to the manufacturer’s protocol. Then the cells were stained for intracellular cytokines with 1 µg/ml of IFN-γ-PE:Cy7, tumor necrosis factor (TNF)-α:PE and interleukin (IL)-2:APC (all from eBioscience) for 30 minutes at 4°C in the dark. Following, the cells were acquired on a FACSCanto and gated according to single cells (forward scatter (FSC)-H vs. FSC-A), lymphocytes (FSC-A vs. side scatter (SSC)-A), CD4 or CD8 expression and then the frequency of cytokine-expressing cells that were CD44^hi^ were determined for both the CD4 and CD8-positive populations. A positive response was defined as an increase in fluorescence intensity above the level of unstained cells. Boolean gating was used to determine the frequency of cells expressing all three cytokines. For detection of antigen-specific CD8 T cells in blood, PBMCs were purified from blood one week after the final immunization by density-gradient centrifugation using Lympholyte Mammal (Cedarlane, Burlington, NC, USA) according to the manufacturer’s instructions. 1×10^6^ cells were stained in V-bottom 96-well plates with 5 µl/well H2-K^b^ AMQMLKETI-PE pentamer (ProImmune, Sarasota, FL, USA) and subsequently with CD19:PE-Cy7, CD8:PerCP-Cy5.5, CD4:APC-Cy7, CD62L:FITC and CD44:APC. After staining the cells were acquired on a FACSCanto and gated according to single cells, lymphocytes, lack of CD19 expression to reduce background noise, CD4 and CD8 expression and then the frequency of CD44^hi^ AMQMLKETI^+^ cells was determined for the CD8^+^ population. All flow cytometry analyses were done using the FlowJo software (TreeStar, Ashland, OR, USA).

#### In vivo cytotoxicity assay

Three weeks after the final immunization, mice were injected intravenously (i.v.) with 4×10^6^ splenocytes from naïve mice of which 50% had been previously stained with 10 µM carboxyfluorescein succinimidyl ester (CFSE, Invitrogen) (termed CFSE^lo^) and the other 50% with 100 µM CFSE (termed CFSE^hi^) and also pulsed for 1.5 hours with 10 µg/ml AMQMLKETI peptide. After 1 day the mice were killed and the percentage of CFSE^hi^ cells (antigen-pulsed) and CFSE^lo^ cells (unpulsed) out the total CFSE^+^ cells in the spleens were determined by flow cytometry. Specific lysis was calculated as follows:
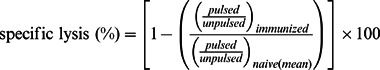



Thus, first the ratio of unpulsed CFSE^lo^ to antigen-pulsed CFSE^hi^ cells was calculated for each animal. Then the ratio was averaged for the naïve group and the specific lysis was calculated as the percentage of each immunized mouse relative to the mean of the naïve mice.

#### Enzyme-linked immunosorbent spot (ELISPOT) assay

The IFN-γ ELISPOT assay was performed according to the manufacturer’s (Mabtech AB, Sweden) protocol. Briefly, ten days after the final immunization splenocytes (either freshly isolated or previously frozen) at final concentration of 125,000 cells/well in triplicates were re-stimulated with pools of peptides set up as a matrix (Table 1) or individual peptides at a concentration of 10 µg/ml during 18 hours of incubation. As positive control staphylococcal enterotoxin B (SEB; Sigma-Aldrich) with a final concentration of 1 µg/ml was included. Numbers of specific IFN-γ-secreting cells were measured in an ELISPOT reader (Autoimmun Diagnostika GmbH, Strassburg, Germany), analyzed with AiD3.1 S.R software and expressed as numbers of spot-forming units (SFU) after background subtraction. If this background-corrected value was negative it has been represented in the graphs and in statistical analyses with the value zero.

#### Statistics

Statistical analyses of differences in expression of intracellular cytokines by CD4 and CD8 T cells and levels of secreted cytokines in response to stimulation with different concentrations of antigen were done by two-way analysis of variance (ANOVA) at a 0.05 significance level followed by the Bonferroni post test. Statistical differences in the levels of specific lysis were determined by one-way ANOVA at the 0.05 significance level followed by Tukey’s post test. An increase in the level of SFU when comparing the OLGa approach to full protein was determined by one-tailed t test and the *P* values have been indicated. ELISPOT positivity was defined as statistically significant by the conservative Distribution Free Resampling (DFR(2×)) statistical test which requires at least a twofold increase between background and experimental means for the difference to be considered significant and thus ensures a low false positive responder rate [25]. Differences in the numbers of responders between groups was determined by Fischer’s Exact test. All analyses, except for DFR(2×), were done using GraphPad Prism (GraphPad Software, La Jolla, CA, USA).

## Results

### Gag p24 in CAF05 Induces High CD4 and CD8 T-cell Responses

**Figure 1:**
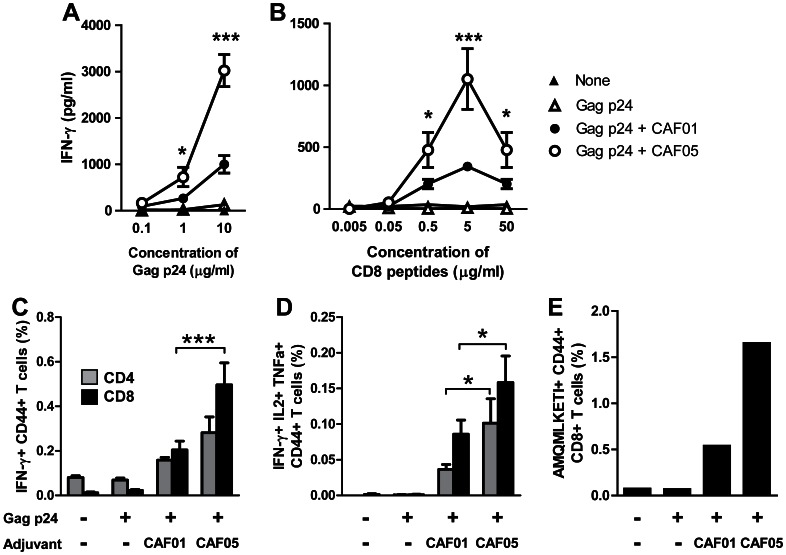
Gag p24 formulated with CAF05 induces antigen-specific CD4 and CD8 T-cell responses in CB6F1 mice. Mice were immunized three times i.p. with 20 µg Gag p24 protein alone (open triangles) or adjuvanted in CAF01 (black circles) or CAF05 (open circles). Naïve mice were used as a negative control (black triangles). The T-cell response in spleens was determined three weeks after the last immunization by restimulation *in vitro* with either whole Gag p24 protein (A), the CD8 T-cell epitope AMQMLKETI-containing peptides 16–17 (B) or 10 µg/ml of a mix of overlapping Gag p24 peptides (C-D). T-cell responses were assessed by ELISA to determine the release of IFN-γ in supernatants after 4 days (A-B) or flow cytometry to determine the percentage of IFN-γ^+^ CD44^hi^ cells of the total CD4 (grey bars) or CD8 (black bars) T cell population (C) and the percentage of cells expressing both IFN- γ, TNF-a and IL-2 after 8 hours of stimulation (D). (A-D) Each data point represents the mean (n = 6) +/− SEM. Where the CAF05 adjuvant induced a statistically significantly higher response than CAF01 determined by two-way ANOVA and the Bonferroni post test, this has been indicated: *p<0.05, ***p<0.001. (E) The percentage of AMQMLKETI-specific CD44^hi^ cells of the total CD8 T-cell population was also determined in blood pooled within each group 1 week after the last vaccination.

**Figure 2:**
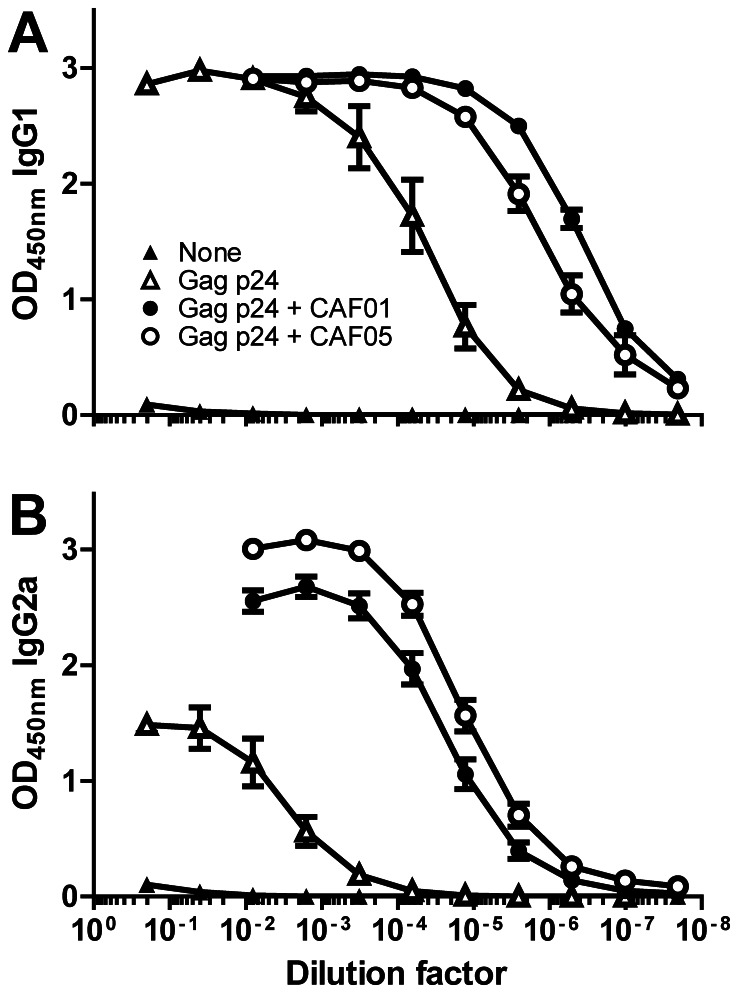
CAF05 enhances Gag p24 specific antibodies to the same level as CAF01. Mice were immunized three times i.p. with 20 µg Gag p24 protein alone (open triangles) or adjuvanted in either CAF01 (black circles) or CAF05 (open circles). Naïve mice were used as a negative control (black triangles). Three weeks after the final immunization, antigen-specific IgG1 (A) and IgG2a (B) antibody responses in serum from individual mice were determined ELISA. Each data point represents the mean (n = 6) +/− SEM.

To make an HIV vaccine which could induce robust CD8 T-cell responses against Gag p24 we produced a recombinant Gag p24 protein based on the consensus sequence of HIV-1 clade B and formulated this in either the CAF01 or CAF05 adjuvant. Thus, CB6F1 mice were injected intraperitoneally (i.p.) three times with two-week intervals in a standard animal immunization experiment and the vaccine recall response was measured three weeks after the last injection by restimulation of the splenocytes with different concentrations of the antigen *in vitro*. The antigen-specific response was measured as the release of IFN-γ after restimulation for 4 days with either whole Gag p24 antigen (Figure 1A) or a mix of two 15-mer peptides containing the known dominant H2-K^d^ CD8 T-cell epitope AMQMLKETI (Figure 1B). Both adjuvanted vaccines induced a good Gag p24-specific IFN-γ release but the CAF05 adjuvant was significantly better than CAF01 when the cells were restimulated with antigen concentrations of 0.5 µg/ml or higher. At the highest peptide concentration the response decreased; this was most likely due to either overstimulation of the cells or inherent toxicity of the peptides. The T-cell responses were further investigated by flow cytometry after 8 hours of restimulation with an overlapping Gag p24 peptide mix at 10 µg/ml. We found that the frequency of total IFN-γ producing CD8 T cells, but not CD4 T cells, was significantly higher when using the CAF05 adjuvant compared to CAF01 (Figure 1C). However, when multifunctional triple-cytokine-producing IFN-γ^+^ IL-2^+^ TNF-α^+^ T cells were assessed, CAF05 was able to induce this potent memory phenotype for both CD4 and CD8 T cells (Figure 1D). The enhanced CD8 T-cell response induced by CAF05 was also reflected in an enhanced frequency of antigen-specific CD8 T cells in the blood one week after the last vaccination (Figure 1E). In contrast to the ability of CAF05 to enhance CD8 T-cell responses, serum IgG1 and IgG2a anti-Gag p24 antibody levels were comparable to those induced by CAF01 (Figure 2).

### Gag p24 in CAF05 Induces Specific Cytotoxicity in vivo

**Figure 3:**
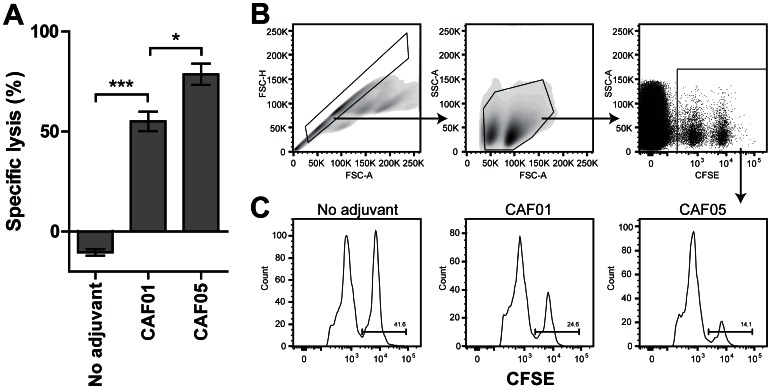
The CAF05 adjuvant enhances the cytotoxicity of CD8 T cells. Mice were immunized three times i.p. with 20 µg Gag p24 protein alone (‘no adjuvant’) or adjuvanted in either CAF01 or CAF05. Three weeks later *in vivo* cytotoxicity was determined 1 day after adoptive transfer of AMQMLKETI-pulsed (CFSE^hi^) or unpulsed (CFSE^lo^) CFSE-stained target cells from naïve mice. (A) The specific lysis relative to the naïve control group. Bars represent the mean specific lysis of individual mice within each group (n = 3/4+/− SEM). Significant differences as determined by one-way ANOVA followed by Tukey’s post test have been indicated: *p<0.05, ***p<0.001. (B) Gating strategy for one representative mouse. (C) Representative histograms of CFSE^lo^ and CFSE^hi^ populations for all three immunization groups.

Having obtained the highest frequency of CD8 T cells when using the CAF05 adjuvant, we next investigated their cytotoxic capacity. Thus, we immunized mice three times with Gag p24 and CAF01 or CAF05 adjuvant as described above. At day 21 after the last immunization we pulsed splenocytes from naïve animals with the minimal CD8 T-cell epitope (AMQMLKETI) and a high concentration of CFSE (Ag^+^ CFSE^hi^) or without antigen and a low concentration of CFSE (Ag^−^ CFSE^lo^). These CFSE^hi^ and CFSE^lo^ cells were mixed in a 1∶1 ratio and ∼ 4 x 10^6^ cells were transferred to each immunized mouse. One day later the degree of cytotoxicity was determined as the specific lysis (Figure 3). Despite a relatively low fraction of antigen-specific cells as determined by ICS (Figure 1 C-D), we observed a high specific lysis and found that the mice immunized with Gag p24 in CAF05 were able to kill a significantly higher proportion of the target cells compared to those immunized with antigen alone or adjuvanted in CAF01. The degree of cytotoxicity was a little lower in the mice immunized with antigen alone (Figure 3, ‘no adjuvant’) compared to that of naïve mice (data not shown) and the specific lysis was therefore negative for this group. Some unspecific lysis is commonly observed in the CFSE^hi^ cells as an effect of the inherent cytotoxic effect of the CFSE dye at high concentrations.

### Antigen Recognition in an HLA-A2/DR Transgenic Mouse

**Figure 4:**
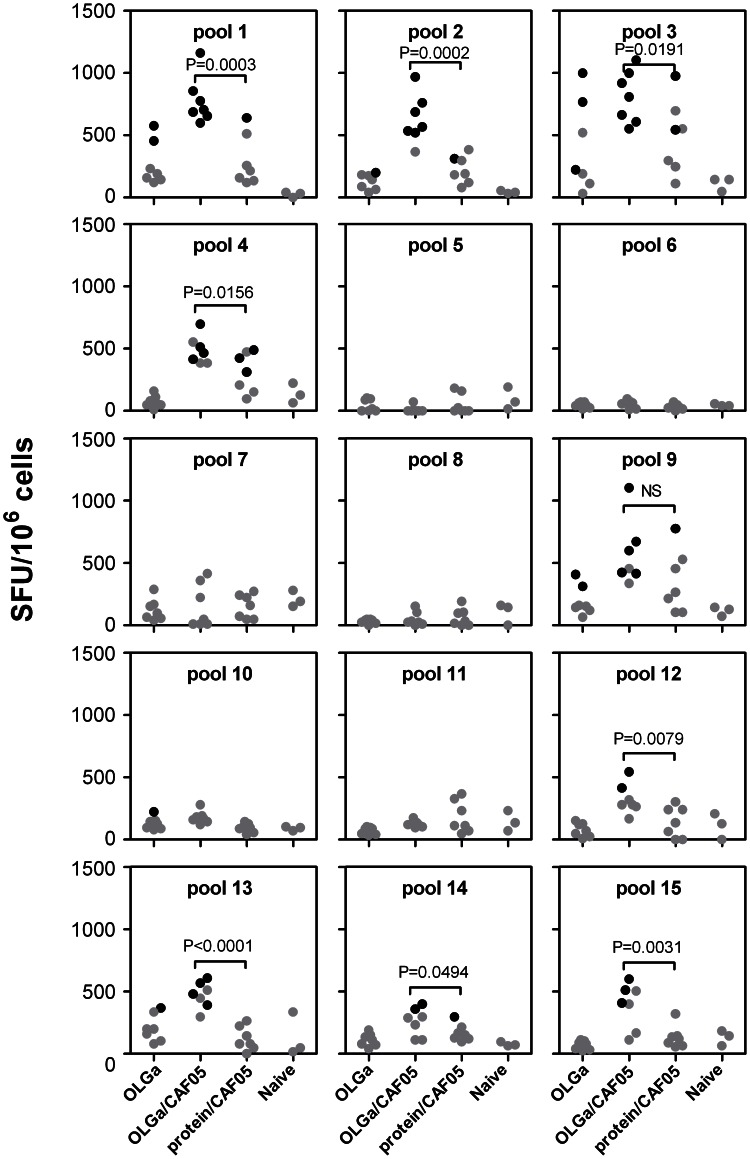
Fragmentation of Gag p24 broadens the T-cell repertoire. HLA-A2/DR-transgenic mice were immunized three times with either the full set of overlapping peptides (OLGa, 2 µg/peptide) with or without CAF05 or protein (5 µg) in CAF05. Naïve mice where used as the negative control. 10 days after the last immunization, splenocytes from individual mice were restimulated with the indicated peptide pool (see Table 1) and the frequency of IFN-γ-producing cells was determined by ELISPOT. Shown are values after background subtraction. Black symbols indicate a significant response for that individual mice as determined by the DFR(x2) method. If a significant response was detected in either the protein/CAF05 or peptides/CAF05 group, statistically significant differences in SFU-levels between these two groups only were determined based on a one-tailed t test and *P*-values are indicated. NS, not significant.

The CAF05 adjuvant was clearly superior as an adjuvant for a Gag p24 vaccine with respect to the induction of both higher CD4 and CD8 T-cell responses and cytotoxicity *in vivo*. Therefore, we decided to use the CAF05 adjuvant and a set of 54 overlapping Gag p24 peptides (15-mers overlapping with 11 amino acids, one peptide was omitted due to solubility problems) in an attempt to broaden the T-cell response and target multiple epitopes compared to immunization with the whole Gag p24 protein. In order to compare the specificity of induced T-cell responses to known epitopes targeted during HIV-1 infection, we used the humanized HLA-A2/DR transgenic mouse strain. The mice were immunized as described in the previous studies and specific T-cell responses were evaluated using the sensitive IFN-γ ELISPOT assay ten days after the last immunization. The 54 overlapping Gag p24 peptides were divided into 15 pools and set up as a matrix so that each peptide was present in two different pools (Table 1). This approach makes it possible to determine the specific peptide response without the need for stimulation with single peptides. Statistically significant responders (indicated by black dots in Figure 4) as determined by the conservative DFR(x2) method [25] were detected after immunization with both overlapping peptides in CAF05 and/or whole protein in CAF05 after restimulation with Pool 1, 2, 3, 4, 9, 12, 13, 14 and 15 (Figure 4). Comparing the significantly responding pools with the matrix, the following individual peptides were possible T-cell targets: 1, 2, 3, 4, 25, 26, 27, 28, 33, 34, 35, 36, 41, 42, 43, 49, 50, 51, 52. Importantly, in all pools with significant responders, except for pool 9, the average frequencies were statistically significantly higher using the overlapping peptides in CAF05 compared to protein in CAF05 (*p*-values are indicated in Figure 4). In addition, there were significantly more individual mice responding in the group that had been immunized with overlapping peptides in CAF05 as compared to the group that had been immunized with whole protein in CAF05 for pool 1 (*p = *0.0047), pool 2 (*p = *0.0291) and pool 3 (*p = *0.021) as determined by Fischer’s Exact test (Figure 4). The statistical analysis comparing significant positive responders in the 2 groups is rather weak due to a low number of mice (n = 7) per group. Thus, despite 4 significant positive responders in the OLGa/CAF05 group and none in the protein/CAF05 group for pool 13, the difference in the number of significant positive responders is not statistically significant (*p = *0.0699).

### Identification of Responding Peptides and Epitopes

**Figure 5:**
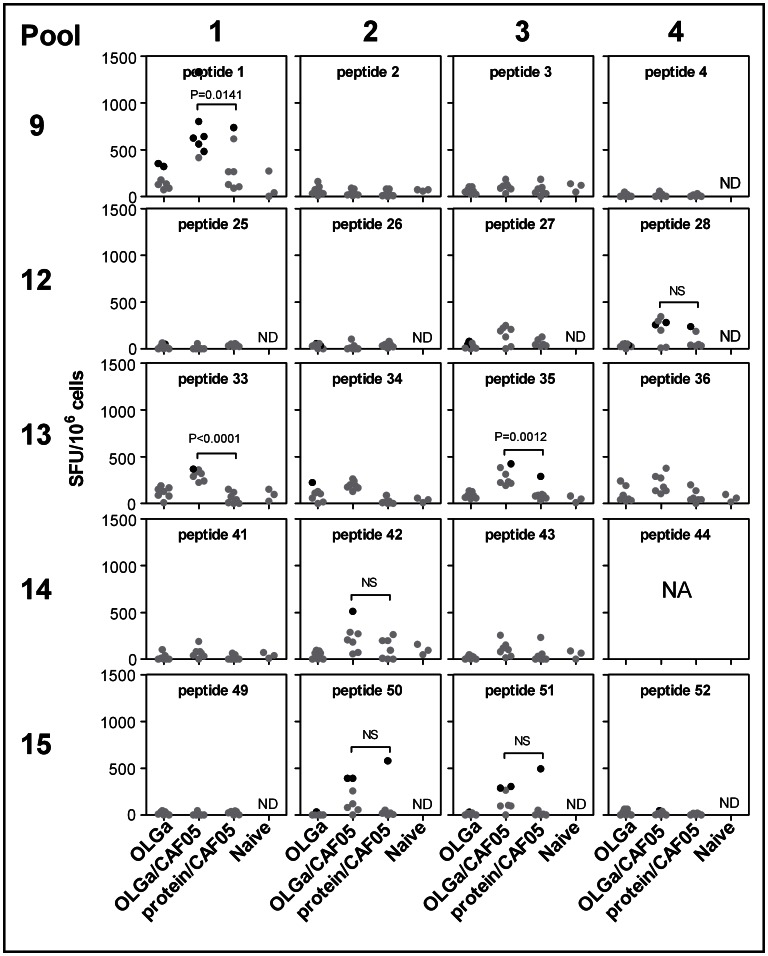
Stimulation of PBMCs with individual peptides selected based on positive peptide pools. HLA-A2/DR-transgenic mice were immunized three times with either the full set of overlapping peptides (OLGa, 2 µg/peptide) with or without CAF05 or protein (5 µg) in CAF05. Naïve mice where used as the negative control. 10 days after the last immunization, splenocytes from individual mice were restimulated with the indicated peptides and the frequency of IFN-γ-producing cells was determined by ELISPOT. Shown are values after background subtraction. Black symbols indicate a significant response for that individual mice as determined by the DFR(x2) method. If a significant response was detected in either the protein/CAF05 or peptides/CAF05 group, statistically significant differences in SFU-levels between these groups were determined based on a one-tailed t test and *P*-values are indicated. NS, not significant. ND, not done. NA, not applicable (peptide 44 could not be dissolved).

We next evaluated the response to the individual peptides identified by the peptide matrix. We found that the peptide strategy induced at least one significant T-cell responder (indicated by black dots in Figure 5) within the peptide/CAF05 group, as identified by the DFR(x2) method [25], for 7 of the 20 peptides: 1, 28, 33, 35, 42, 50, 51 (Figure 5). In addition, the frequency of responding cells in the peptide/CAF05 group was significantly increased compared to immunization with whole protein/CAF05 for 3 of these peptides: 1, 33, 35, whereas the number of significant positive responders was significantly higher only for peptide 1 (*p = *0.0291).

**Figure 6:**
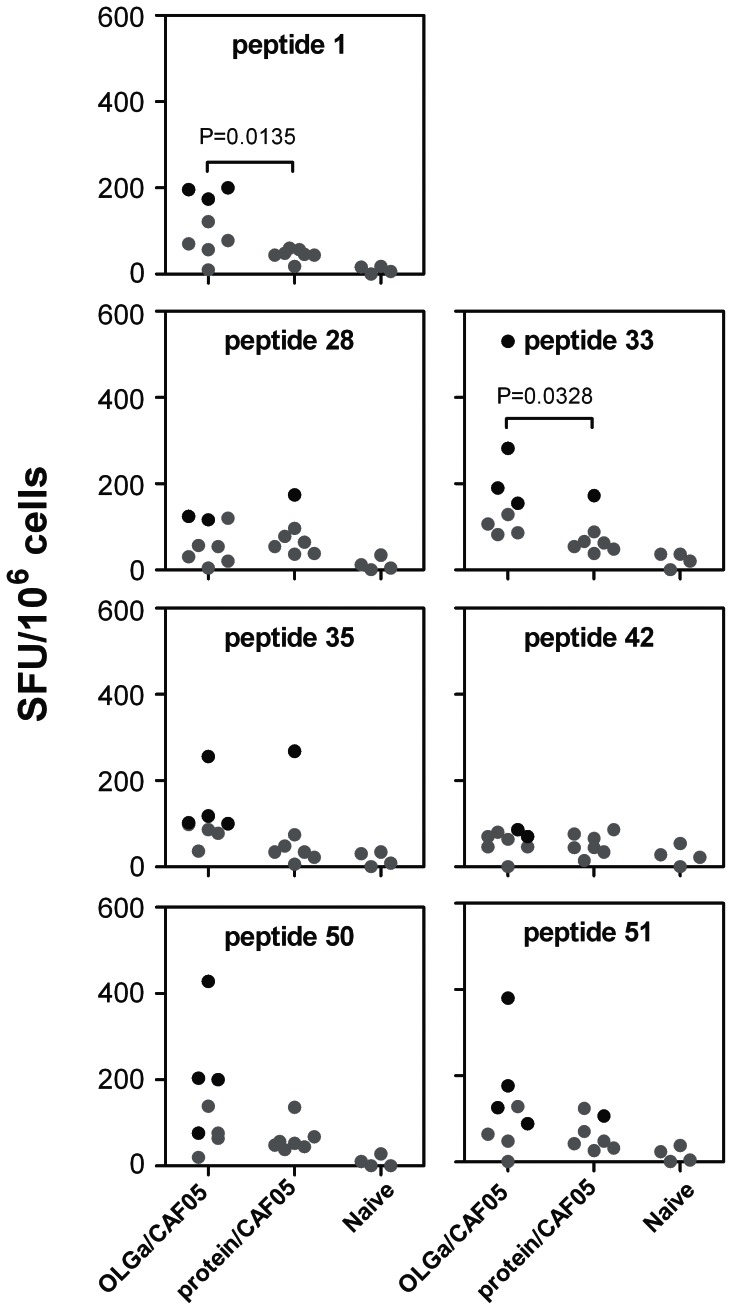
T-cell recognition of peptides after immunization with equal molar doses of peptide sequences. HLA-A2/DR-transgenic mice were immunized three times with either the full set of overlapping peptides (OLGa, 2 µg/peptide) in CAF05 or protein (60 µg) in CAF05. Naïve mice where used as the negative control. 10 days after the last immunization, splenocytes from individual mice were restimulated with the indicated peptides and the frequency of IFN-γ-producing cells was determined by ELISPOT. Shown are values after background subtraction. Black symbols indicate a significant response for that individual mice as determined by the DFR(x2) method. If a significant response was detected in either the protein/CAF05 or peptides/CAF05 group, statistically significant differences in SFU-levels between these groups were determined based on a one-tailed t test and *P*-values are indicated. NS, not significant.

To confirm these findings we repeated the experiment but used a 12 times higher protein dose to ascertain that the increased response induced by the overlapping peptides was not due to a suboptimal protein dose. In the previous studies we had used a lower dose of protein that did not contain the same molar quantities of epitopes as was present in the peptide vaccines because short peptides are more sensitive to degradation *in vivo* compared to whole protein and, therefore, their epitope concentrations cannot be directly compared. In this experiment we used similar molar quantities and tested the response to the 7 peptides that were identified as positive in order to investigate if the peptide strategy was indeed superior to using whole protein. In this experiment (Figure 6) the SFU-levels were lower than observed in the previous experiment (Figure 5) but as we have commonly observed similar variations between different experiments using this mouse strain, this was ascribed to biological and interexperimental variation. For all the 7 peptides, we found at least 2 significant positive responders (indicated by black dots in Figure 6) after peptide vaccination and for 2 peptides (peptide 1 and 33) the level of this response was significantly higher than after protein vaccination (Figure 6). Nevertheless, the number of responders was not significantly different between the two vaccination approaches. However, we did find that protein vaccination only induced significant responders for 4 of the 7 peptides (peptide 28, 33, 35 and 51) and in all 4 cases there was not more than one significant positive responder (Figure 6). This confirms our previous findings that the peptide approach was superior with regards to broadening of the T-cell repertoire.

### Predictions of CD4 and CD8 T-cell Epitopes within Positive Peptides

As the ELISPOT assay chosen does not discriminate between CD4 and CD8 T-cell responses, we identified possible CD4 and CD8 epitopes within the responding peptides using the NetMHC-IIpan server [26] and the HLArestrictor server [27], respectively (Table 2). Besides predicting known DRB1*0101-binding CD4 T-cell epitopes within all 7 peptides, HLA-A0201-binding CD8 T-cell epitopes were also predicted in all peptides except for peptide 42. Despite generally lower MHC-I than MHC-II-binding affinities, two of the predicted CD8 T-cell epitopes had been previously described: IILGLNKI/WIILGLNKI in peptide 33 and RMYSPTSI/RMYSPTSIL within peptide 35. The HLA-A02∶01-binding epitopes, QMVHQAI in peptide 1, TLQEQIGWMT in peptide 28 and ALGPAATL/ALGPAATLEEM within the overlapping peptides 50–51 are not reported in the Los Alamos HIV Molecular Immunology Database [28]. Whether the low response to peptide 28 was a response to the predicted MHC-I epitope TLQEQIGWMT or the known MHC-II epitope contained within this sequence (LQEQIGWMT) cannot be deduced from our study but their binding affinities are at a similarly low level. All remaining peptides contained CD4 T-cell epitopes with a high MHC-II binding affinity and lower MHC-I binding affinity. Whether the use of the CD8 T-cell inducing CAF05 adjuvant had enhanced the CD8 T-cell responses or not is therefore impossible to determine from our study. Thus, the overlapping peptide vaccine approach lead to a broadened T-cell response but further studies will be required to decipher the exact type of response in detail.

**Table 2:** Identification of CD4 and CD8 T-cell epitopes within responding 15-mer peptides.

Peptide no.	15-mer sequence	HLArestrictor 1.2^1^			NetMHCIIpan-2.1^3^		
		HLA-A02∶01 peptide	Affinity (nM)	Known epitope^2^	DRB1*0101 binding core	Affinity (nM)	Known epitope^2^
**1**	PIVQNLQGQMVHQAI	QMVHQAI	685	no	VQNLQGQMV	8	Yes^4^
**28**	STLQEQIGWMTNNPP	TLQEQIGWMT	659	no	LQEQIGWMT	537	Yes^5^
**33**	IYKRWIILGLNKIVR	IILGLNKI	571	Yes^6^	WIILGLNKI	14	Yes^7^
		WIILGLNKI	580	Yes^8^			
**35**	GLNKIVRMYSPTSIL	RMYSPTSI	100	Yes^9^	IVRMYSPTS	2	Yes^10^
		RMYSPTSIL	173	Yes^11^			
**42**	VDRFYKTLRAEQASQ	–			YKTLRAEQA	3	Yes^12^
**50**	DCKTILKALGPAATL	ALGPAATL	227	no	ILKALGPAA	2	Yes^13^
**51**	ILKALGPAATLEEMM	ALGPAATL	227	no	ILKALGPAA	4	Yes^13^
		ALGPAATLEEM	372	no			

1 reference [27],

2 reference [28],

3 reference [26],

4 reference [48],

5 reference [49],

6 reference [50],

7 reference [51],

8 reference [52],

9 reference [53],

10 reference [54],

11 reference [55],

12 reference [56],

13 reference [57].

## Discussion

To meet the high diversity of HIV-1, a broad epitope T-cell response, which includes both CD4- and CD8 T cells, seems to be required. To address this challenge we have compared a standard subunit vaccine approach with whole Gag p24 protein as the antigen with a fragmented antigen approach. We used overlapping 15-mer peptides spanning the entire Gag p24 and to achieve a good mixed T-cell response, we formulated the antigens in a novel cationic liposome adjuvant, CAF05 which induce both CD4 and CD8 T-cell responses. This adjuvant is based on the CAF01 adjuvant [24], [29], which comprises the cationic amphiphile DDA and the synthetic mycobacterial cord factor TDB, formulated with the TLR3-ligand, Poly(I:C), favoring cross presentation of the vaccine antigen [21], [22], [30], [31]. CAF05 also induces robust antibody responses at the same level as CAF01. In CB6F1 mice we achieved strong IFN-γ production by both CD8 and CD4 T cells after vaccination with Gag p24 in CAF05 and high cytotoxicity towards antigen-pulsed target cells *in vivo*. This is consistent with earlier results showing that adding Poly(I:C) to CAF01 primes CD8^+^ T cells to efficiently lyse target cells and reduce tumor growth in different tumor vaccine mouse models [21]. Furthermore, the increased fraction of multifunctional IFN-γ^+^ TNF-α^+^ IL-2^+^ T cells indicates a high quality response with good effector function and high long-term memory potential [32].

For our fragmented antigen approach we therefore focused on this optimal CTL adjuvant, CAF05, and found that immunization of humanized HLA-A2/DR-transgenic mice with OLGa induced an increased and broader T-cell immune response compared to the full-length protein. An increased T-cell repertoire by fractionation of the immunogen is in agreement with Li *et al* [33] who found a broadening of the T-cell response to DNA vaccine HIV-1 Gag epitopes if fractionated into p17, p24, and p7. Furthermore, when p24, which contains the most T-cell epitopes, was further fractionated into three pieces the response was broadened even more [33]. They found this broadening effect to be restricted to the boosting immunogen rather than the priming immunogen [33]. It has also been shown that dendritic cells transduced with adenovirus serotype 5 vectors expressing SIV Gag divided into 7 fragments primes a broader T-cell response *in vitro* than those expressing full-length Gag [34]. In addition, removing the dominant epitope from the early secretory antigenic target (ESAT)-6 protein from *Mycobacterium tuberculosis* or immunizing with individual smaller overlapping fragments in combination with the cationic CAF01 adjuvant, have been shown to expose subdominant and cryptic T-cell epitopes that are not recognized in mice neither during tuberculosis infection nor after immunization with full length ESAT-6 protein in CAF01 [35]. In contrast, immunizing macaques with aldrithiol-2 inactivated whole SIV-pulsed autologous blood cells (IPAL) induced Gag-specific CD4 T-cell responses as good as, or better, than those achieved with overlapping peptides from SIV Gag (OPAL) [10], [15]. However, no comparison of whole SIV Gag protein versus fractionated SIV Gag was made, so these data do not directly contradict a fragmented vaccine approach.

An important consideration when comparing full-length protein with overlapping peptides is the difficulty in assuring similar amounts of available epitopes for MHC presentation. Thus, small peptides are more prone to extracellular degradation [36], may not contain the signaling sequences required for correct processing and presentation of the epitope on MHC molecules [37], [38], and unknown T-cell epitopes may not be represented in full length within the chosen peptide sequence frame. We have attempted to address this by comparing the OLGa strategy with protein given in both a low standard dose and a high dose representing equal molar quantities of the epitopes. This led to less statistically significant differences when using the high dose protein but the trend was nevertheless the same: fragmentation broadened the T-cell repertoire.

The ELISPOT assay that was used to assess T-cell responses in HLA-A2/DR-transgenic mice does not discriminate between CD4 and CD8 T cells. Therefore, several intracellular cytokine stainings of these cells were done but these analyses were consistently non-interpretable (data not shown). The explanation is most likely the inherently low numbers of CD8 T cells in this transgenic mouse strain [23] which would also suggest that the majority of the responses observed was derived from CD4 T cells. Nevertheless, as all peptides besides one (peptide 42) contained predicted epitopes for both T-cell subsets, it remains to be elucidated whether the OLGa approach broadened the CD4 or CD8 T-cell repertoire or both.

Another challenge to overcome when seeking a broad T-cell response is immune focusing. During infection and vaccination the T-cell immune response often focuses towards only a few immunodominant epitopes [39], [40], [41]. Similarly, candidate HIV-1 vaccines evaluated in human clinical trials have mainly induced immunodominant epitopes [42]. Several mechanisms are involved in immunodominance including processing, affinity between the peptide fragment and MHC molecules, access to antigen-presenting cells and availability of specific T cells [43], [44]. Thus, physical separation of antigens targeting different antigen-presenting cells increases the breadth of T-cell responses [45], [46] but immune competition between epitopes may still be a limiting factor [47]. In our study we have used overlapping 15-mer peptides to maximize fragmentation and the physical separation of the antigens for different APCs when using only one injection site and we demonstrate a convenient broadening of HIV-1 Gag p24 epitope responses. Loading of individual peptides onto separate liposomal adjuvant particles to increase physical separation of the antigens even further has been attempted for other antigens without success (unpublished observations).

In order to identify if both dominant and subdominant or cryptic epitopes were targeted following vaccination with OLGa, we used the HLA-A2/DR-transgenic mouse model as the human HLA-A2 restricted HIV-1 epitopes have been extensively characterized. Although only two new possible HLA-A2 restricted epitopes, and no HLA-DRB1*0101 restricted epitopes, were identified, the T-cell response included both known immune subdominant and dominant epitopes (Table 2). However, some immune competition may still have occurred as several known HLA-A2 epitopes were not recognized in our experiments. Immunization with individual peptides could be used to show if the lack of responses to some epitopes was a result of immune competition or related to the experimental design, such as the specific peptide sequences which could negatively affect peptide processing for presentation or make the peptides more prone to extracellular degradation [37], [38].

In conclusion, we found that when we used overlapping peptides as the vaccine antigen in CAF05 a broader T-cell response was achieved compared to when whole Gag p24 protein was used as the antigen. Thus, a fragment vaccine strategy with broadening of Gag responses may help to meet the high HIV-1 viral diversity.
